# The current state and future perspectives of cannabinoids in cancer biology

**DOI:** 10.1002/cam4.1312

**Published:** 2018-02-23

**Authors:** Paweł Śledziński, Joanna Zeyland, Ryszard Słomski, Agnieszka Nowak

**Affiliations:** ^1^ Department of Biochemistry and Biotechnology Poznań University of Life Sciences Dojazd Street 11 60‐632 Poznań Poland; ^2^ Institute of Human Genetics of the Polish Academy of Sciences Strzeszyńska Street 32 60‐479 Poznań Poland

**Keywords:** Cancer, Cannabidiol, Cannabinoids, Cannabis, CBD, Tetrahydrocannabinol, THC

## Abstract

To date, cannabinoids have been allowed in the palliative medicine due to their analgesic and antiemetic effects, but increasing number of preclinical studies indicates their anticancer properties. Cannabinoids exhibit their action by a modulation of the signaling pathways crucial in the control of cell proliferation and survival. Many in vitro and in vivo experiments have shown that cannabinoids inhibit proliferation of cancer cells, stimulate autophagy and apoptosis, and have also a potential to inhibit angiogenesis and metastasis. In this review, we present an actual state of knowledge regarding molecular mechanisms of cannabinoids’ anticancer action, but we discuss also aspects that are still not fully understood such as the role of the endocannabinoid system in a carcinogenesis, the impact of cannabinoids on the immune system in the context of cancer development, or the cases of a stimulation of cancer cells’ proliferation by cannabinoids. The review includes also a summary of currently ongoing clinical trials evaluating the safety and efficacy of cannabinoids as anticancer agents.

## Introduction

Nowadays, we observe an increasing public and scientific interest in the medical applications of *Cannabis* plants. In the USA, marijuana is now allowed for medical applications in 24 states and the District of Columbia. Hemp oil which has low concentration of Δ^9^‐tetrahydrocannabinol is allowed in 16 subsequent states 1, 2. Furthermore, during the last decade, we have collected a great pool of evidence from preclinical and clinical studies that *Cannabis* and cannabinoids have a therapeutic potential in many medical fields, and can even display some anticancer characteristics.

### Cannabinoids

Cannabinoids are lipophilic ligands for specific cell‐surface cannabinoid receptors (CB). This class of molecules can be divided into three main groups: phytocannabinoids, endocannabinoids, and synthetic cannabinoids. Phytocannabinoids are secondary metabolites of *Cannabis* plants. About 100 phytocannabinoids have been described, of which Δ^9^‐tetrahydrocannabinol (THC) is main psychoactive compound. Action of THC in human organism relies on mimicking endogenous agonists of CB receptors—endocannabinoids 3. It is responsible for euphoria and has analgesic, antiemetic, and anti‐inflammatory properties, however, its psychoactivity strongly limits medical potential 4. Another phytocannabinoid which gains medical attention is cannabidiol (CBD). It has low affinity for cannabinoid receptors and acts independently of them. CBD interacts with other receptors such as transient receptor potential channel subfamily V member 1 (TRPV1), orphan G‐protein coupled receptor (GPR55), or peroxisome proliferator‐activated receptors (PPARs). They have been proposed to be classified as CB receptors, but their exact role in endocannabinoid signaling is still under discussion 3. CBD has anxiolytic properties and attenuates THC psychoactive effects 4.

Endocannabinoids are part of the endocannabinoid system, which is composed of cannabinoid receptors, their endogenous ligands, and the enzymes involved in their metabolism. The most widely studied endocannabinoids are anandamide (AEA) and arachidonoylglycerol (2‐AG). To date, two cannabinoid receptors (CB1 and CB2) have been identified in mammalian tissues 5, 6. They belong to G‐protein coupled receptor family 3. The activation of each of them leads to an inhibition of adenylyl cyclase via G proteins (G_i/o_), which in turn activates many metabolic pathways such as mitogen‐activated protein kinase pathway (MAPK), phosphoinositide 3‐kinase pathway (PI3K), cyclooxygenase‐2 pathway (COX‐2), accumulation of ceramide, modulation of protein kinase B (Akt), and ion channels 3, 7. Most of the cannabinoids’ effects in neural and nonneural tissues rely on activation of CB1 receptor. Its high expression has been observed in these areas of central nervous system, that are engaged in the modulation of motor behavior, memory, learning, emotions, perception, and endocrine functions 3, 8. Studies using CB1 knockout mice suggest that this receptor plays a role also in behavioral disorders such as depression, anxiety, feeding, or cognition 9. CB2 receptors have been found in immune cells, but their presence was revealed also in nervous system 10, 11. In addition to its neuromodulatory function, endocannabinoid system has been shown to play other important functions such as control of the energy metabolism, immunity, cardiovascular tone, and reproduction 12, 13.

According to the contribution of the endocannabinoid system in a regulation of such variety of processes, its pharmacological modulation becomes promising therapeutic strategy. To date, cannabinoids have been exploited in the palliative medicine. In the USA there are two cannabinoid‐based drugs approved for use by US FDA: nabilone and dronabinol. Nabilone (THC synthetic analogue) is allowed for the treatment of nausea and vomiting induced by chemotherapy, and sleep disorders. Dronabinol (synthetic THC) is approved also for nausea and vomiting due to chemotherapy and for the treatment of weight loss associated with AIDS. Another drug approved outside the USA (Austria, Canada, Czech Republic, Demark, France, Germany, Italy, Poland, Spain, Sweden, United Kingdom) is nabiximols (Sativex^®^, oromucosal spray, THC and CBD in 1:101 ratio), which is allowed for a treatment of spasticity associated with multiple sclerosis. Medical *Cannabis* in the form of marijuana (dried flowers and leafs) is illegal in the USA at the federal level according to the Controlled Substances Act 1970 as a Schedule I substance, but some states have legalized it for medical purposes 14.

Besides palliative properties of cannabinoids, it has been shown in wide range of in vitro and animal models, that they also exhibit anticancer effects 7, 15, 16, 17.

### Endocannabinoid system and cancer

Despite numerous studies conducted during the last decade, there are still inconsistent data regarding the exact role of cannabinoid system in cancer development. The upregulated expression of CB receptors and the elevated levels of endocannabinoids have been observed in a variety of cancer cells (skin, prostate, and colon cancer, hepatocellular carcinoma, endometrial sarcoma, glioblastoma multiforme, meningioma and pituitary adenoma, Hodgkin lymphoma, chemically induced hepatocarcinoma, mantel cell lymphoma), but it is not always correlated with the expression level of these receptors in tissue of origin 7, 11, 18, 19. Furthermore, concentration of endocannabinoids, expression level of their receptors, and the enzymes involved in their metabolism frequently are associated with an aggressiveness of cancer. This implies that an overactivation of endocannabinoid system might be protumorigenic and plays an essential role in the development of cancer 20, 21. It has been shown that absence of CB receptors leads to decrease in ultraviolet‐light skin carcinogenesis in murine model 22. In agreement with this, CB2 receptor contributes to human epidermal growth factor receptor (HER2) pro‐oncogenic signaling and an overexpression of CB2 increases susceptibility for leukemia development after leukemia viral infection 23, 24.

On the other hand, there are reports indicating that an activation of the cannabinoid receptors can impair cancer development and hence endocannabinoid signaling can be antitumorigenic. This idea is supported by reports showing that endocannabinoid‐degrading enzymes are upregulated in cancer cell lines and in human tumors 25, 26. Furthermore, it has been shown that silenced expression of CB1 receptor leads to an acceleration of intestinal adenoma growth, whereas activation of this receptor attenuates its growth in murine model 27. Tumor growth rate has been observed to decrease after a reduction in the expression of endocannabinoid‐degrading enzyme. The elevated level of endocannabinoids has been shown to reduce the development of precancerous lesions in mouse colon 28. Endocannabinoids have been demonstrated to inhibit the growth of prostate cancer cells in dose‐dependent manner 29.

### Anticancer effects of cannabinoids

The first report of antiproliferative properties of cannabinoids comes from 1975, when Munson et al. demonstrated that Δ^9^‐tetrahydrocannabinol inhibits lung adenocarcinoma cell growth of in vitro cell line and in murine model after oral administration 30. Many cannabinoids, ranging from phytocannabinoids (THC, CBD), endocannabinoids (2‐arachidonoylglycerol, anandamide), to synthetic cannabinoids (JWH‐133, WIN‐55,212‐2), have shown ability to inhibit proliferation, metastasis, and angiogenesis in a variety of models of cancer 7, 8. On the other hand, there are reports that have indicated that under certain circumstances, cannabinoids can be protumorigenic 31, 32, 33.

**Figure 1:**
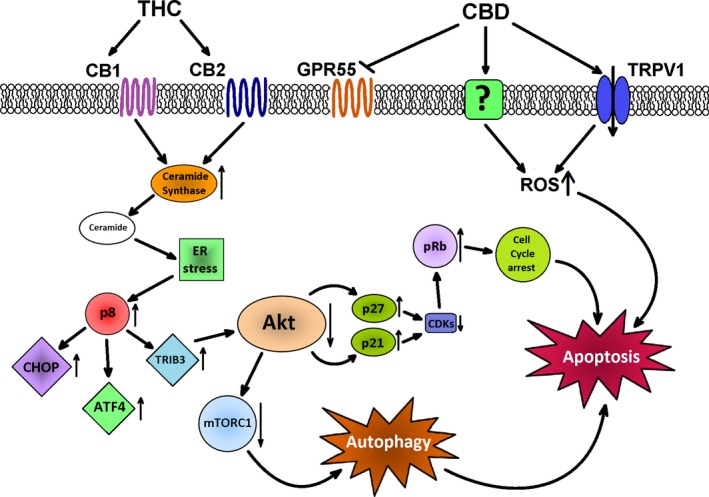
The known mechanisms responsible for the induction of apoptosis by cannabinoids. CBD, cannabidiol; THC, Δ^9^‐tetrahydrocannabinol; CB1, cannabinoid receptor 1; CB2, cannabinoid receptor 2; TRPV1, receptor potential channel subfamily V member 1; GPR55, orphan G‐protein coupled receptor 55; ROS, reactive oxygen species; ER, endoplasmic reticulum; p8, protein p8 (Nuclear Protein 1, NUPR1); CHOP, CCAAT/‐enhancer‐binding protein homologous protein; ATF4, activating transcription factor 4; TRIB3, tribbles pseudokinase 3; Akt, protein kinase B; mTORC1, mammalian target of rapamycin C1; p21, cyclin‐dependent kinase inhibitor 1; p27, cyclin‐dependent kinase inhibitor 1B; CDK, cyclin‐dependent kinase; pRb, retinoblastoma protein.

Despite some inconsistent data, the main effect of cannabinoids in a tumor is the inhibition of cancer cells’ proliferation and induction of cancer cell death by apoptosis. It has been shown that CB1 and CB2 receptor agonists stimulate apoptotic cell death in glioma cells by induction of de novo synthesis of ceramide, sphingolipid with proapoptotic activity 34, 35. Accumulation of ceramide leads to an activation of endoplasmic reticulum (ER) stress‐related signaling pathway. This involves enhanced expression of stress‐regulated protein p8 (Nupr1), transcriptional regulator, taking part in a control of tumor development, as well as its downstream targets, activating transcription factor 4 (ATF4), C/EBP homologous protein (CHOP), and Tribbles homolog 3 (TRIB3) (Fig. 1) 36, 37. Various detrimental stimuli, such as an oxidative stress, Ca^2+^ depletion, viral infection, or some anticancer agents may also activate ER stress as a cellular response 38. Effects of this complex phenomenon include the translation arrest, degradation of misfolded proteins and restoration of ER protein‐folding capacity. If this response fails, ER stress can lead to activation of intrinsic apoptosis pathway 38. Indeed, activation of p8‐regulated pathway causes inhibition of prosurvival protein kinase B (Akt) by TRIB3, that in turn leads to an inhibition of the mammalian target of rapamycin complex 1 (mTORC1) and eventually to autophagy‐mediated cell death (Fig. 1) 39, 40.

It has been demonstrated that process of autophagy is upstream of apoptosis in mechanism of cell death induced by cannabinoids. An inhibition of autophagy prevents apoptosis induced by cannabinoids, while an inhibition of apoptosis prevents only cell death but not the autophagy 39, 41, 42, 43.

It has been shown that cannabinoids induce process of autophagy in cancer cell lines such as glioma, melanoma, hepatic, and pancreatic cancer 39, 41, 42, 43. Moreover, some additional mechanisms have been demonstrated to contribute to the process of an induction of cell death by cannabinoids in certain cell lines. In hepatocellular carcinoma, ER stress induced by cannabinoids can lead to an activation of AMP‐activated protein kinase and calcium/calmodulin‐dependent protein kinase kinase 2, that in turn acts as an additional factor in autophagy‐mediated cell death 41. In breast carcinoma and melanoma, cannabinoid‐stimulated inhibition of Akt leads to the activation of cyclin‐dependent kinase inhibitory proteins p21 and p27, causing phosphorylation of the retinoblastoma protein and eventually cell cycle arrest and apoptosis 44, 45, 46. In glioma, downregulation of Akt signaling can lead to reduction in phosphorylation of Bcl‐2‐associated death promoter (BAD), proapoptotic Bcl‐2 family member, resulting in apoptosis 47. Similar results have been obtained in prostate carcinoma cells (Table S1) 48.

Cannabinoids devoid of psychoactive properties also exhibit anticancer potential. They do not affect CB receptors directly and their exact mechanism of action is still not fully elucidated. To date, the most frequently proposed mechanism of their action relies on the stimulation of production of reactive oxygen species (ROS), which leads to autophagy‐mediated apoptotic cell death 17, 49, 50, 51. Another interesting explanation is that CBD can prevent the degradation of anandamide (AEA) and subsequently leads to increased endocannabinoid concentration by acting as an inhibitor of fatty acid amide hydrolase (FAAH) 52, 53. This notion raises the possibility that the observed actions of CBD can be, in fact, partially the result of an elevated level of AEA. These observations are in line with the described earlier relations between endocannabinoids and cancer development.

Most of the research implicates that the action of CBD and other cannabinoids devoid of psychoactive properties is not linked to a direct activation of the CB receptors. It has been proposed that interactions of CBD with other types of receptors (GPR55, TRPV1, TRPM8) can play a significant role in its action, for example, cannabidiol and cannabigerol (CBG) exhibit their anticancer activity, acting as potent antagonists of TRPM8 receptors 3, 54. However, there are reports suggesting that CBD can induce apoptosis in cancer cells partially via direct or indirect activation of CB2 receptor 55. Recent studies have shown that CBD reduces cancer cell viability in many cancer types such as neuroblastoma, glioblastoma, melanoma, leukemia, colorectal, breast, lung, or prostate cancer (Table S1) 41, 50, 51, 54, 56, 57, 58, 59.

### Cannabinoids and the immune system

The mechanism of the immunomodulatory effects of cannabinoids is still not fully elucidated. Research has been focused mainly on the CB2 receptor, mostly due to its expression primarily in cells of the immune system. CB1 receptors have been noticed in the T lymphocytes and it is proposed that their activation may be connected with the cytokine biasing induced by cannabinoids 60. The highest level of CB2 expression has been observed in B cells, followed by NK cells, monocytes, polymorphonuclear neutrophils, and T cells 61. It has been shown that the expression level of CB2 correlates with the cell activation state and with the presence of immune modulators 62.

The immune system is postulated to be involved in the control of growth and development of many types of cancer. One of the key regulators of the antitumor immune response is cytokines profile. IL‐2 and IFN‐γ are cytokines that promote a Th1 response, whereas IL‐4 and IL‐5 promote a Th2 response. Additionally, IL‐10 has been linked to suppression of the Th1 response. It is postulated that a Th1 response is crucial for an effective immune response against many tumors 63.

The results of the CB2 receptors’ activation include changes in the cytokine release from immune cells 64. Phytocannabinoids with high affinity for CB2 receptors, such as THC, exhibit modulatory effects on both cellular and humoral immunity. THC action was linked to the inhibition of IFN‐γ production, change in Th1/Th2 profile, and suppression of T‐cell proliferation 65, 66. Nonpsychotropic cannabinoids with low affinity for CB receptors have also been proven to exhibit immunomodulatory action. CBD shows anti‐inflammatory effects by antagonizing CB receptors agonists and subsequently leading to the inhibition of immune cell migration 67. Most of the studies indicate that cannabinoids exhibit immunosuppressive action 68.

The most extensively examined immunomodulatory effects of cannabinoids in context of cancer are regarding the changes in the activity of T cells. As mentioned above, cannabinoids can influence T‐cell proliferation and reduce their cytolytic activity, but according to research conducted to date, their most significant effects include the modulation of T helper cell subsets (Th1/Th2) and TGF‐β secretion 62, 69. The action of cannabinoids leads to the bias in the subtypes of the Th cells from the proinflammatory Th1 profile to the anti‐inflammatory Th2 profile. This includes an increase in the production of Th2‐promoting cytokines (IL‐10 and TGF‐β) and a decrease in the production of Th1 cytokines (IL‐2, IL‐12, and IFN‐γ), as well as a decrease in the expression of the IFN‐γ, IL‐12, and IL‐12 receptors 68. IL‐10 and TGF‐β have been demonstrated to inhibit host immunity and may interfere with antitumor immune responses 31, 32. Proposed explanatory mechanism of the Th biasing includes the difference in the expression level of CB receptors between Th subtypes and antigen‐presenting cells and the fact that the increase in the production of Th2‐promoting cytokines can itself lead to the inhibition of Th1 cells 62. Moreover, Δ9‐THC treatment resulted in upregulation of Th2 cytokine genes and downregulation of Th1 cytokine genes, effects associated with histone modifications 70. It has also been observed that cannabinoids can lead to the B‐cell immunoglobulin class switching from IgM to IgE and consequently biasing toward Th2‐type immunity in CB2 receptor‐dependent manner 71. It has been proven that CB receptor ligands can lead to the suppression of the production of the proinflammatory cytokines (TNF‐α, IL‐1, IL‐2, IL‐6, IL‐12) 62.

Another mechanism that may be involved in cannabinoids’ immunosuppressive action is the upregulation of the regulatory T cells (Tregs) which leads to the inhibition of the immune responses 72. It has also been proposed that cannabinoids can affect T cells by the induction of apoptosis 73, 74. Another possibility is that cannabinoids effects on immune cells are at least partially induced indirectly via other suppressive mechanisms such as release of cortisone 68. The effects on the Th17 cells subsets have not been fully described to date. CBD, cannabinoid with low affinity to CB receptors, has been shown to suppress T cells function and inhibit IL‐2 production. Interestingly, CB receptors seem to take part in the modulation of those phenomena 75.

Indeed, there are reports indicating the suppression of anticancer immune response by THC. It has been demonstrated that THC suppresses host immune reactivity against cancer in murine lung cancer model (Lewis lung carcinoma, 3LL and line 1 alveolar cell carcinoma L1C2), leading to the increase in the tumor growth 32. The authors have observed an increase in the immune inhibitory cytokines (IL‐10 and TGF‐β) and a decrease in IFN‐γ. Moreover, injection of anti‐IL‐10‐ or anti‐TGF‐β Abs counteracted the THC‐induced acceleration in tumor growth. CB2 receptors antagonists also blocked the effects of THC administration. Interestingly, L1C2 tumor growth in immunodeficient SCID (Severe Combined Immunodeficiency) mice treated with THC remained unaffected, suggesting that THC‐induced suppression of antitumor response is based on the enhancement of the activity of the host immunosuppressive networks 32.

Similar results were obtained in the study of mouse mammary carcinoma. It has been demonstrated that THC exposure leads to the significant increase in the 4T1 carcinoma growth and metastasis due to the inhibition of the specific antitumor immune response 31. Observed effects include an increase in the production of IL‐4 and IL‐10 cytokines suggesting the shift from Th1 to Th2 profile. The authors have subsequently demonstrated elevated expression of Th2‐related genes and the downregulation of a number of Th1‐related genes. Finally, they have shown that an administration of the anti‐IL‐4 and anti‐IL‐10 Abs led to a partial reversion in the Δ9‐THC‐induced suppression of the immune response to 4T1. Observed effects were mediated by CB2 receptors 31.

It is possible that tumors originating from tissues of low CB receptors expression would be significantly less sensitive to cannabinoids anticancer action and, eventually, due to THC immunosuppressive properties, such tumors may find a favorable environment for growth and development. It is also possible that anticancer properties of cannabinoids may be compensated by their immunosuppressive action, finally leading to promotion of the tumor growth. The possible enhancement of the tumor growth due to THC‐mediated impairment in antitumor immune responses may be a strong disadvantage in the medicinal use of THC in patients with cancer.

On the other hand, Haustein et al. has demonstrated that an important part of cannabinoids’ anticancer action may be constituted by the modulation of the expression of the intercellular adhesion molecule 1 (ICAM‐1) on lung cancer cells 76. THC, CBD, and R(+)‐methanandamide (an endocannabinoid analogue) have been shown to promote the expression of ICAM‐1 on lung cancer cell lines A549, H460, and metastatic cells derived from a lung cancer patient. It led to the enhancement of the cancer cells susceptibility to adhere to lymphokine‐activated killer LAK cells and to subsequent lysis. Furthermore, lysis mediated by the LAK cells was not observed in noncancerous bronchial epithelial cells, BEAS‐2B 76.

Another interesting part of cannabinoids’ action regards their proposed anti‐inflammatory activity. Chronic inflammation has been associated with the development of neoplasia; therefore, reducing inflammation may, to some extent, contribute to the prevention of carcinogenesis. Indeed, immunocompetent rats administered with THC for 2 years have shown a decrease in tumor incidence and increase in overall survival rate 77. It has been proposed that observed effects can be related to anti‐inflammatory properties of THC 78, 79.

### Selectivity and stimulation of viability

Viability of noncancerous cells seems to remain unchanged or sometimes even elevated by cannabinoids 34, 35, 36, 39, 80. On the other hand, cannabinoids can trigger apoptotic cell death in some types of nontransformed cells, especially those of high proliferative properties such as endothelial cells 81. The cellular response to cannabinoids relies on different mechanisms in cancerous and noncancerous cells. Furthermore, proapoptotic properties of cannabinoids are connected with an activation of either CB1 and/or CB2 receptors in some cell lines (glioma), but in other types of cell (breast, pancreatic, hepatic carcinoma) it seems to rely only on activation of CB2 receptor 15.

The aspect of cannabinoids’ action that requires consideration is their concentration‐dependent activity. It has been demonstrated in vitro that cannabinoids can exhibit a stimulatory activity in nanomolar concentration and an inhibitory activity in micromolar concentration (biphasic response), which significantly exceeds concentrations usually detected in blood of marijuana smokers 62. Studies in vitro have shown that submicromolar concentrations of CB receptor agonists cause increase in the proliferation of certain cancer cell lines and that this effect was dependent on an activity of ADAM17 metalloprotease, an activation of epidermal growth factor receptor (EGFR), and subsequent stimulation of extracellular signal‐regulated kinases (ERK) and Akt pathways 33. Concentration of THC used in described experiment corresponded to its serum concentration obtained by smoking or oral administration of THC 33.

### Inhibition of angiogenesis and metastasis

Besides the above described proapoptotic effect in cancer cells, cannabinoids exhibit some other important and potentially valuable properties. It has been demonstrated that they can inhibit angiogenesis by blocking an activation of the vascular endothelial growth factor (VEGF) pathway. Cannabinoids lead to the downregulation of vascular endothelial growth factor itself and its receptors (VEGFR1 and VEGFR2) in glioma, skin, and thyroid carcinoma 82, 83, 84. Moreover, cannabinoids can suppress tumor‐induced endothelial cell proliferation and as mentioned earlier, induce their apoptosis, thus impair development of tumor vasculature 81, 85. These observations are in agreement with the experiments indicating that a pharmacological blockade of ceramide biosynthesis leads to an abrogation of cannabinoid‐induced inhibition of VEGF production and VEGFR‐2 activation 83.

Cannabinoids have also been shown to reduce spontaneous and induced metastases in animal models and to inhibit an invasiveness of cancer cells in vitro (breast, lung, cervical cancer, and glioma) 86, 87, 88, 89, 90. These effects are partially connected with a modulation of the activity of extracellular proteases and their inhibitors 86, 90. The pharmacological inhibition of ceramide biosynthesis and the expression of p8 protein lead to the prevention of the mentioned effects 86.

The studies conducted to date indicate that antiangiogenic and antimetastatic characteristics of CB receptor agonists, similar to their antiproliferative effects, rely on the stimulation of ceramide biosynthesis and a modulation of pathways involving p8 protein. Cannabinoids that are not agonists of CB receptors (CBD), have also been shown to exhibit such properties. The effects of CBD have been connected to downregulation of an expression of Id‐1, an inhibitor of basic helix–loop–helix transcription factors, which has been shown to be a key regulator of the metastatic potential of breast cancer 91.

It has been also demonstrated that CBD can lead to a decrease in lung tumor cell invasion and metastasis via mechanism relied on the upregulation of the intercellular adhesion molecule 1 (ICAM‐1). Induction of ICAM‐1 has been linked to TIMP‐1 (tissue inhibitor of matrix metalloproteinases‐1) ‐dependent anti‐invasive action of cannabinoids 92.

Another interesting observation is that the inhibitors of fatty acid amide hydrolase (FAAH), namely arachidonoyl serotonin and URB597, also exhibit TIMP‐1‐dependent anti‐invasive properties. Increased levels of FAAH substrates (e.g., AEA, 2‐AG) in lung cancer cells incubated with FAAH inhibitors have been demonstrated 93.

### Anticancer effects of cannabinoids in clinical trials

Data collected to date regarding anticancer effects of cannabinoids are almost completely limited to preclinical studies conducted on cell lines and animal models. The first experiment that was conducted on human subjects was a pilot clinical study on nine terminal patients with recurrent glioblastoma who were resistant to the standard therapy 19. Patients received THC intratumorally. This way of administration was safe and patients did not exhibit any overt psychoactive effects. In some patients the tumor growth rate decreased. Changes observed upon THC administration in two patients can be connected with anticancer effect of THC according to previous preclinical studies (decreased cell proliferation, occurrence of apoptosis) 19. Despite these interesting observations, it is not possible to draw significant conclusions from the study on a group of nine. This shows a need for further clinical trials, which could help to assess the dosage and the potential interaction of cannabinoids with other substances.

There are some new clinical trials, which aim to assess the safety and the impact of cannabinoids or cannabinoid‐based preparations on the cancer patients. These studies are currently ongoing or have ended recently, but the results have not been published to date. First of them is a study on the safety of nabiximols in combination with temozolomide in patients with recurrent glioblastoma (NCT01812603, NCT01812616) 94, 95. The aim of the second trial is to evaluate the impact of CBD as a single treatment in patients with solid tumors (NCT02255292) 96. There are also studies assessing the safety and effects of dexanabinol (synthetic cannabinoid) in patients with solid tumors and brain cancer, and in healthy subjects (NCT01489826, NCT01654497, NCT02054754) 97, 98, 99.

## Conclusions

*Cannabis* plants produce a substantial amount of cannabinoids and other secondary metabolites. The cannabis‐based preparations can exhibit their effects by synergistic interactions between their various components, that is, changes in a bioavailability or in cellular transport, an activation or a deactivation of other metabolites, multi‐target effects of synergistic partners and other 100. It has been demonstrated that extracts of *Cannabis* exhibit stronger effects on the subjects with spasticity than pure THC 101. Some cannabinoids have been demonstrated to attenuate psychoactive effects of THC or smoked marijuana 13, 102. Other metabolites of *Cannabis* such as terpenes or flavonoids can significantly contribute to a modulation of cannabinoids’ pharmacokinetics 100. Pure cannabinoids are more convenient for study and to a subsequent standardization as a medical preparation, but still *Cannabis* extracts with specified amounts of cannabinoids seem to be valuable aim for further studies, also as potential anticancer agents.

An interesting idea is a combination of cannabinoids with conventional anticancer drugs, which can exhibit synergistic potential. The promising results from studies on animal models of glioblastoma treated with THC and temozolomide have led to, mentioned above, clinical trial of this chemotherapeutic agent and Sativex 94, 103. Similar observations from the study on pancreatic adenocarcinoma showed that gemcitabine administered with cannabinoids synergistically inhibited cancer cell growth 104.

To date, *Cannabis* or its preparations have found an application in a palliative medicine due to its analgesic and antiemetic effects, an attenuation of the side effects of chemotherapy or a capacity to treat spasticity in multiple sclerosis. Some cannabinoids‐based drugs have been registered in several countries as mentioned earlier (e.g., nabiximols, dronabinol, nabilone); however, recent meta‐analysis of the results from clinical trials involving *Cannabis* and cannabinoid‐based drugs demonstrates existing moderate‐ or low‐quality evidence supporting the use of these agents in a treatment. In the case of chronic pain and spasticity, there were moderate‐quality evidences supporting the use of cannabinoids. Low‐quality proofs were found to support Cannabis use in treatment of nusea/vomiting due to chemotherapy, weight loss in AIDS, sleep disorders and Tourette syndrome 14.

We are still initial stages of incorporating *Cannabis* products in the clinical care. There is still a lack of profound safety and efficacy clinical trials and it is very difficult or even impossible to assess the potential benefits and risk of using cannabinoids in many cases. Many aspects wait for an elucidation: the ways of administration, dosages, interactions with other drugs, or assessing adverse effects. The most common way of using recreational marijuana is smoking, which is unsuitable way of an administration from a medical point of view. Approved cannabinoids‐based drugs have form of oromucosal spray or capsules. Another important issue is the lack of easy accessible biomarkers showing the responsiveness of patients to a cannabinoid treatment.

Cannabinoids show antitumor activity in cell lines and in animal models of cancer, but we still do not have data concerning their efficacy and safety from well‐prepared clinical trials. Moreover, antitumor effects of cannabinoids have to overcome their known immunosuppressive effects which can be potentially protumorigenic. The interactions between cannabinoids and classical cytotoxic agents have to be precisely defined. These observations lead us to the conclusion, that further profound studies are doubtlessly needed to verify the idea of introducing cannabinoids into the cancer treatment.

## Conflict of Interest

The authors declare that they have no conflict of interest.

## Supporting information


**Table S1**. Overview of cannabinoids’ actions in cancer cell lines.
