# The current state and future perspectives of cannabinoids in cancer biology

**DOI:** 10.1002/cam4.1876

**Published:** 2018-11-21

Paweł Śledziński, Joanna Zeyland, Ryszard Słomski, Agnieszka Nowak. Cancer Medicine 2018;7(3):765‐775

In the published version of this article,^1^ third paragraph under Cannabinoids heading, the authors wrote: “Sativex^®^, oromucosal spray, THC and CBD in 1:101 ratio,” whereas it should have been written: “Sativex^®^, oromucosal spray, THC and CBD in a ~1:1 ratio.”

The authors apologize for this error.
