# Supplementary material for: The current state and future perspectives of cannabinoids in cancer biology
Source: Cancer Med. 2018 Feb 23;7(3):765–75. doi: 10.1002/cam4.1312 (PMC5852356; doi:10.1002/cam4.1312)
Supplement: Supplementary file 1 — Table S1. Overview of cannabinoids’ actions in cancer cell lines. [file CAM4-7-765-s001.doc]

Table 1. Overview of cannabinoids’ actions in cancer cell lines.

| Compound | Type of cancer | Observed changes | Reference |
| --- | --- | --- | --- |
| AEA  2-AG  Methanandamide (AM-356) | Prostate adenocarcinoma   - PC3   Primary adenocarcinoma cells | - Decrease in the viability of the cells - Cell cycle arrest - Apoptosis - Increase in the level of active caspase-3 - Decrease in the level of Bcl-2 - Increase in the level of ERK - Decrease in the level of Akt | Orellana-Serradell et al. 2015 |
| THC | Breast adenocarcinoma   - MDA-MB-231   Invasive ductal carcinoma   - MCF-7   Mouse mammary carcinoma   - 4T1 | - No decrease in the viability of the 4T1 and MCF-7 cells - Increase in the growth of the 4T1 tumor *in vivo* - Increase in the metastasis of the 4T1 tumor *in vivo* - No effects in the immunodeficient mice - Alteration in the cytokine profile | McKallip et al. 2005 |
| THC | Murine Lewis lung carcinoma   - 3LL   Murine alveolar cell carcinoma   - L1C2 | - Increase in the growth of the 3LL and L1C2 tumors *in vivo* - No effects in the immunodeficient mice - Alteration in the cytokine profile | Zhu et al. 2000 |
| AEA  THC  HU-210  Win55,212-2 | Glioblastoma   - U373-MG   Lung carcinoma   - NCI-H292   Squamous cell carcinoma   - SCC-9   Bladder carcinoma   - 5637 | - EGFR activation - Mediation of EGFR transactivation by cannabinoid-induced cleavage of proAR and/or proHB-EGF by TACE/ADAM17 - Increase in the EGFR- and metalloprotease-dependent cancer cells proliferation | Hart et al. 2004 |
| THC | Astrocytoma  - U87MG | - Induction of autophagy via upregulation of p8 - Reduction of cannabinoid-induced cell death by pharmacological inhibition of autophagy - Inhibition of Akt and mTORC1 - Apoptotic cell death promoted by THC-induced autophagy | Salazar et al. 2009 |
| THC  CBD | Melanoma   - CHL-1 - A375 - SK-MEL-28 | - Decrease in the viability of the THC treated cells - Autophagy and apoptosis in THC treated cells - Reduction in the growth of tumors treated by THC and THC+CBD *in vivo* | Armstrong et al. 2015 |
| THC  JWH-133  WIN 55,212-2 | Pancreatic adenocarcinoma   - MiaPaCa2 - Panc1 - Capan2 - BxPc3 | - Decrease in the viability of the cells - Increase in the ceramide level - Induction of apoptosis by CB2 receptor activation - Upregulation of p8 - Upregulation of ATF4 and TRIB3 - Reduction in the growth of tumors *in vivo* | Carracedo et al. 2006 |
| THC  JWH-015 | Hepatocellular carcinoma   - HepG2 - HuH-7 | - Decrease in the viability of the cells - Effects relied on activation of CB2 receptor - Autophagy mediated cell death - Autophagy relied on TRIB3 upregulation, inhibition of Akt/mTORC1 and AMPK stimulation - Reduction in the growth of tumors *in vivo* | Vara et al. 2011 |
| THC | Breast carcinoma   - EVSA-T   Breast adenocarcinoma   - MDA-MB-231 - MDA-MB-468 - SKBR3   Invasive ductal carcinoma   - MCF-7 - T-47D | - Decrease in the proliferation of cells - Effects relied on activation of CB2 receptor - Arrest of the cell cycle at the G2-M transition via down-regulation of Cdc2 - Induction of apoptosis | Caffarel et al. 2006 |
| THC  JWH-133  WIN-55,212–2 | Melanoma   - A375 (human) - MelJuso (human) - B16 (mouse) | - Decrease in the viability of the cells - Cell cycle arrest at the G1-S transition via inhibition of Akt and hypophosphorylation of pRb - Reduction in the growth of tumors *in vivo* - Decrease in the formation of metastases - Decrease in the proliferation of cells *in vivo* - Increase in the apoptotic activity in *vivo* | Blázquez et al. 2006 |
| THC | Breast carcinoma   - EVSA-T | - Upregulation of JunD expression - Antiproliferative effects of THC relied on the JunD activity | Caffarel et al. 2008 |
| WIN 55,212-2  WIN 55,212-3 | Rat C6 glioma   - C6 | - Decrease in the viability of the cells - Cell cycle arrest at G1/G0 phase - Downregulation of the Akt and ERK - Decrease in the phosphorylated Bad - Mitochondrial depolarization - Activation of caspase cascade | Ellert-Miklaszewska et al. 2005 |
| WIN-55,212-2 | Prostate carcinoma   - LNCaP - PC3 | - Cell cycle arrest at G1/G0 phase - Increase in the expression of p53 and p27/KIP1 - Decrease in the expression of cyclins: D1, D2, E - Decrease in the expression of cdk-2, -4, -6 - Decrease in the expression of pRb - Downregulation of E2F (1-4) - Decrease in the expression of DP1 and DP2 - Upregulation of ERK1/2 - Inhibition of PI3k/Akt - Increase in Bax/Bcl-2 ratio | Sarfaraz et al. 2006 |
| CBD | Breast adenocarcinoma   - MDA-MB-231 - SKBR3   Invasive ductal carcinoma   - MCF-7 - ZR-75-1 | - Decrease in the viability of the cells - Coexistence of apoptosis and autophagy - Endoplasmic reticulum stress - Inhibition of Akt/mTOR/4EBP1 - Decrease in the expression of cyclin D1 - Mitochondrial depolarization - Translocation of BID to the mitochondria - Release of cytochrome c to the cytosol - Activation of the intrinsic apoptotic pathway - Increase in the generation of reactive oxygen species (ROS) | Shrivastava et al. 2011 |
| CBD | Glioblastoma   - U251   Primary glioma stem cells (GSC) lines | - Decrease in the viability of the cells - Increase in the generation of reactive oxygen species (ROS) - Increase in the survival rate of mice bearing GSC xenografts - Inhibition of the GSC self-renewal - Activation of the p-p38 pathway - Downregulation of Sox2, Id1 and p-STAT3 - Adaptation of a subset of GSC and tumor regrowth - Therapeutic resistance mediated by enhanced expression of xCT and by PN–MES transition | Singer et al. 2015 |
| CBC  CBD  CBG  CBN  CBDA  CBGA  CBDV  CBGV  THC  THCA  THCV  THCVA  BDS (Biological drug substance) - extracts from *Cannabis sativa* L. | Prostate carcinoma   - LNCaP - 22RV1 - DU-145 - PC-3 | - Decrease in the viability of the cells - Increase in the effects of bicalutamide and docetaxel (standard drugs for the treatment of prostate cancer) in the presence of CBD - Reduction of the LNCaP xenograft growth and increase in the effects of bicalutamide and docetaxel against LNCaP and DU-145 xenograft - Activation of the intrinsic apoptotic pathway - Cell cycle arrest at the G1-S transition - Down-regulation of AR, p53 activation and elevation of reactive oxygen species in LNCaP cells | De Petrocellis et al. 2013 |
| THC  CBD | Neuroblastoma   - SK-N-SH - IMR-32 - NUB-6 - LAN-1 | - Decrease in the viability of the cells - Cell cycle arrest at G1/G0 phase - Decrease in the cell invasiveness - Reduction in the growth of tumors *in vivo* | Fisher et al. 2016 |
| CBD | Breast carcinoma   - SUM159 - MDA-MB-231-SCP2 - MVT-1   Murine breast carcinoma   - 4T1.2   Murine leukemia   - RAW264.7 | - Inhibition of the epidermal growth factor (EGF)-induced cell proliferation, migration and invasion - Inhibition of the EGF-induced activation of EGFR, ERK, AKT and NF-kB signaling pathways - Inhibition of the MMP2 and MMP9 secretion - Reduction in the growth of tumors and inhibition of metastasis *in vivo* - Inhibition of the recruitment of tumor-associated macrophages in primary tumor stroma and secondary lung metastases | Elbaz et al. 2014 |
| CBD | Lung adenocarcinoma   - A549   Large cell lung carcinoma   - H460   Primary non-small-cell lung carcinoma cells | - Decrease in the viability of the cells - Upregulation of COX-2 and PPAR-γ expression - Upregulations of PGE_2_, PGD_2_, and 15d-PGJ_2_ - Inhibition of the cannabidiol-induced viability loss by NS-398 (COX-2 inhibitor) and GW9662 (PPAR-γ antagonist) - Inhibition of the cannabidiol-induced viability loss by transfection of cells with COX-2 and PPAR-γ siRNA - Reduction in the growth of tumors *in vivo* - Reduction of the tumor-regressive action of cannabidiol by pretreatment with GW9662 | Ramer et al. 2013 |
| CBD | T acute lymphoblastic leukemia   - Jurkat | - Decrease in the viability of the cells - Resistance of the cells cultured in physiological conditions to CBD (up to 40 μM) - Increase in the number of cells in G1 phase - Inhibition of Akt/mTOR and ribosomal protein S6 | Kalenderoglou et al. 2017 |
| CBG  CBD  CBDV  CBC | Colon adenocarcinoma   - Caco-2 - HCT 116 | - Decrease in the viability of the cells - Promotion of apoptosis - Increase in the generation of reactive oxygen species (ROS) - Upregulation of CHOP expression - Reduction in the growth of tumors *in vivo* | Borrelli et al. 2014 |

15d-PGJ_2_ -15-Deoxy-Delta-12,14-prostaglandin; 4EBP1 - 4E-binding protein 1; ADAM17 - ADAM metallopeptidase domain 17; Akt - protein kinase B; AMPK - adenosine monophosphate-activated kinase; AR - androgen receptor; ATF4 - activating transcription factor 4; Bad - Bcl2-associated agonist of cell death; Bax - bcl-2-like protein 4; Bcl-2 - B-cell lymphoma 2; BID - BH3 interacting-domain death agonist; CBC – cannabichromene; CBD – cannabidiol; CBDA - cannabidiolic acid; CBDV – cannabidivarin; CBG – cannabigerol; CBGA - cannabigerol acid; CBGV – cannabigevarin; CBN – cannabinol; Cdc2 - cyclin-dependent kinase 1; CHOP - C/EBP homologous protein; COX-2 - cyclooxygenase-2; DP1, DP2 - heterodimeric partners of E2F protein family; EGFR - epidermal growth factor receptor; ERK - extracellular signal-regulated kinases; Id1 - inhibitor of DNA binding 1; JunD - transcription factor jun-D; MMP2 - matrix metalloproteinase-2; MMP9 - matrix metallopeptidase 9; mTORC1 - mammalian target of rapamycin C1; NF-kB - nuclear factor kappa-light-chain-enhancer of activated B cells; p27/ KIP1 - cyclin-dependent kinase inhibitor 1B; p53 - tumor protein p53; p8 – protein p8 (Nuclear Protein 1, NUPR1); PGD_2_ - prostaglandin D2; PGE_2_ - prostaglandin E2; PI3K - phosphoinositide 3-kinase; PN–MES transition - upregulation of mesenchymal (MES) markers with concomitant downregulation of proneural (PN) markers; PPAR-γ - peroxisome proliferator-activated receptor gamma; pRb - retinoblastoma protein; proAR – proAmphiregulin; proHB-EGF - proHeparin-binding epidermal growth factor-like growth factor; p-STAT3 - phosphorylated signal transducer and activator of transcription 3; Sox2 - SRY (sex determining region Y)-box 2; TACE - tumor necrosis factor α-converting enzyme; THC - Δ^9^-tetrahydrocannabinol; THCA - tetrahydrocannabinolic acid; THCV - Δ9-tetrahydrocannabivarin; THCVA - Δ9-tetrahydrocannabivarinic acid; TRIB3 - tribbles pseudokinase 3; xCT - antioxidant response system Xc catalytic subunit.
